# Prognostic effect of pregnancy on young female patients with nasopharyngeal carcinoma: results from a matched cohort analysis

**DOI:** 10.18632/oncotarget.8008

**Published:** 2016-03-09

**Authors:** Lu Zhang, Huai Liu, Lin-Quan Tang, Qiu-Yan Chen, Shan-Shan Guo, Li-Ting Liu, Ling Guo, Hao-Yuan Mo, Chong Zhao, Xiang Guo, Ka-Jia Cao, Chao-Nan Qian, Mu-Sheng Zeng, Jian-Yong Shao, Ying Sun, Jun Ma, Ming-Huang Hong, Hai-Qiang Mai

**Affiliations:** ^1^ Sun Yat-Sen University Cancer Center, State Key Laboratory of Oncology in South China, Collaborative Innovation Center for Cancer Medicine, Guangzhou, P. R. China; ^2^ Department of Nasopharyngeal Carcinoma, Sun Yat-Sen University Cancer Center, Guangzhou, P. R. China; ^3^ GCP center, Sun Yat-Sen University Cancer Center, Guangzhou, P. R. China; ^4^ Department of Radiotherapy, Hunan Cancer Hospital, Changsha, P. R. China; ^5^ Department of Radiotherapy, The Affiliated Cancer Hospital of Xiangya School of Medicine, Central South University, Changsha, P. R. China; ^6^ Key Laboratory of Translational Radiation Oncology, Changsha, P. R. China; ^7^ Department of Molecular Diagnostics, Sun Yat-Sen University Cancer Center, Guangzhou, P. R. China; ^8^ Department of Radiation Oncology, Sun Yat-Sen University Cancer Center, Guangzhou, P. R. China

**Keywords:** nasopharyngeal carcinoma, pregnancy, prognosis, survival

## Abstract

**Objectives:**

We aimed to evaluate the prognosis of pregnancy-associated patients with nasopharyngeal carcinoma (NPC) in a young population.

**Methods:**

From June 1999 to December 2010, 51 patients aged ≤ 35 years who were diagnosed with NPC during pregnancy or within one year after delivery were admitted into the pregnancy-associated group in our institution. An additional 51 patients who were not pregnant at diagnosis were selected from 451 patients based on the matching criteria to match the pregnancy-associated female patients. The primary endpoint was overall survival (OS). The secondary endpoints were progression-free survival (PFS) and distant-metastasis failure-free survival (DMFS) and locoregional failure-free survival (LRFS).

**Results:**

The advanced stage was not different between the pregnant and the non-pregnant group before matching (69.8% vs. 70.3%, *P* = 0.690). No difference in OS at the median follow-up time of 92 months was observed between the pregnancy-associated and the non-pregnant group (85.4% vs. 92.2%, *P* = 0.478); likewise, no differences were observed regarding PFS and DMFS. However, the pregnancy-associated group had worse LRFS than the non-pregnant group (84.8% vs. 95.9%, *P* = 0.033). When the pregnancy-associated patients were dichotomized into an early pregnancy group and a late pregnancy group, our data showed that pregnancy interval did not seem to impact the risk of death or relapse.

**Conclusion:**

Our results show that patients in the pregnant group did not seem to have more advanced stage or inferior survival than that in the non-pregnant group.

## INTRODUCTION

Nasopharyngeal carcinoma (NPC) is distinct from other head and neck cancers because of its histology, epidemiology and treatment strategies. It is commonly observed in Southern China but is rarely found in North America. Because NPC is a chemo- and radio-sensitive cancer, patients with early stage NPC will receive radiotherapy alone, and most of those patients are cured. Conversely, patients with advanced stage NPC will receive concurrent chemoradiotherapy, and some of those patients fail because of distant lesions or locoregional recurrence. Some patients are diagnosed with NPC during pregnancy, and no data regarding the incidence of this condition has been reported. Additionally, under the stimulus of pregnancy, NPC disease is particularly aggressive with advanced stage or distant metastasis, and treatment with concomitant chemoradiotherapy has been deemed pointless and unsatisfactory; thus, the survival outcome is poorer among pregnant patients than among non-pregnant patients. Unfortunately, studies about NPC patients during pregnancy are lacking, and evidence-based management of NPC during pregnancy has not been possible because most information is based on a small number of [1–4] studies. Two case-report articles regarding NPC patients during pregnancy have been recorded. The earliest case reported on a woman who was diagnosed with advanced stage NPC (T4N2-3M1) with mediastinal metastases [2]. She underwent four courses of chemotherapy and 2D-RT therapy but died from her disease within 6 months. Thus, from this case, it appeared that NPC patients who were diagnosed during pregnancy had a horrible prognosis. A later case report on a Taiwanese woman was published in 2007. This female patient was diagnosed with stage T4N2M0 and received chemotherapy and IMRT after delivery. Throughout a 3-year follow-up after delivery, she remained progression-free [3]. This case demonstrated that pregnant patients can still have promising outcomes. In addition, a small cohort study including 27 female patients with NPC showed the negative effect of pregnancy on patients with NPC. From these three articles, we could hypothesize that the NPC patients with pregnancy likely had advanced stage NPC, but their survival results varied. More importantly, the small number of cases and case-report studies can lead to increased biases and confusion of cause and effect. Because we did not have enough information to illustrate the effect of pregnancy on patients with NPC, we designed this matched cohort study to explore the prognosis of NPC patients during pregnancy; moreover, the results could provide useful information for pregnant patients when they seek advice.

## RESULTS

### Patient characteristics

**Figure 1:**
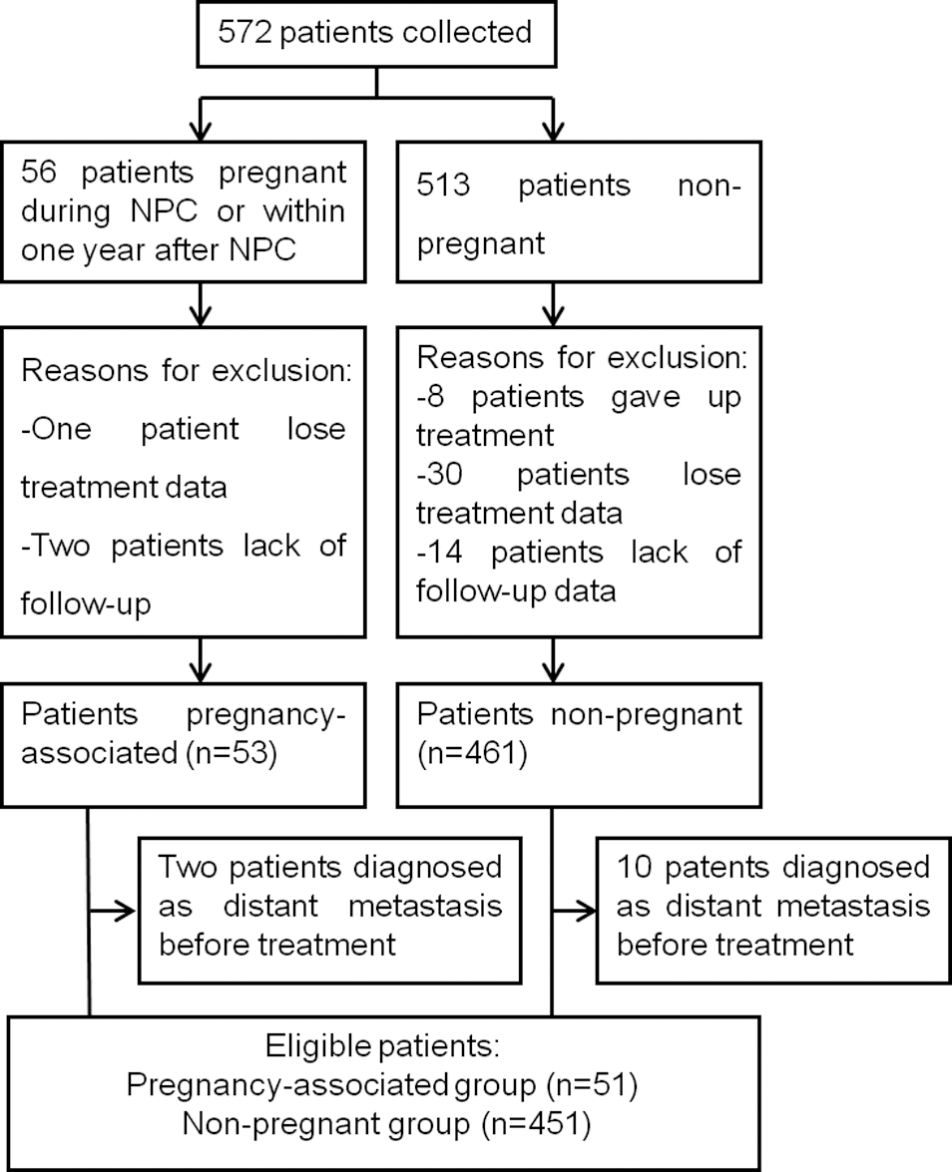
Flowchart summarizing the patients who were eligible for the study

A total of 572 female patients with NPC of WHO I–III who were ≤ 35 years old were enrolled in our institution during April 1999 to December 2010. Of these women, 56 patients became pregnant during or within one year after the completion of pregnancy. After applying the inclusion and exclusion criteria, 68 patients were excluded for various reasons (Figure 1), resulting in 504 patients (51 pregnancy-associated patients and 453 non-pregnant patients) who were eligible for the analyses. The baseline characteristics of the subjects are shown in Table 1. As shown in Table 1, before excluding the patients with distant metastases (DM), all the variables were comparable between the pregnant group and the non-pregnant group except for median age (30 yrs vs. 32 yrs, *P* = 0.044). After the exclusion of patients with DM and the patients who did not fit the matching criteria, the median age for the pregnancy-associated group was 30 years (range, 23–35), and the median age for the non-pregnant group was 31 years (range, 23–35); no significant difference was observed.

**Table 1:** Baseline characteristics

	Pregnant group (including DM) (%)	Non-pregnant group (including DM) (%)	*P^b^* value	Pregnancy-associated group	Non-pregnant group
**Age (months, median [range])**	30 (23–35)	32 (16–35)	0.044	31 (23–35)	30 (23–35)
**Histology**					
WHO I	4 (7.5)	18 (3.9)	0.373	4 (7.8)	2 (3.0)
WHO II–III	49 (92.5)	443 (96.1)		47 (92.2)	49 (97.0)
**ECOG**					
0–1	51 (96.2)	460 (99.1)	0.369	51 (100.0)	51 (100.0)
2	2 (3.8)	4 (0.9)		0(0.0)	0 (0.0)
**T classification**					
T1–T2	23 (43.4)	200 (43.2)	1.000	22 (43.1)	22 (43.1)
T3–T4	30 (56.6)	263 (56.8)		29 (56.9)	29 (56.9)
**N classification**					
N0–N1	30 (56.4)	231 (49.9)	0.395	30 (58.8)	30 (58.8)
N2–N3	23 (43.4)	232 (50.1)		21 (41.2)	21 (41.2)
**M classification**					
M0	51 (96.2)	453 (97.6)	0.683	0	0
M1	2 (3.8)	11 (2,4)		0	0
**Overall stage**^a^					
I–II	14 (26.4)	128 (27.5)	0.690	14 (27.5)	14 (27.5)
III–IVa–b	37 (69.8)	326 (70.3)		37 (72.5)	37 (72.5)
IVc	2 (3.8)	10 (2.2)		0	0
**RT technique**					
2D-RT	41 (80.4)	354 (80.1)	1.000	41 (80.4)	42 (82.4)
IMRT	10 (19.6)	88 (19.9)		10 (19.6)	9 (17.6)
**RT dose**					
Nasopharynx	70	70	1.000	70	70
Neck	60	60		63	60
**Follow-up time (month, median)**	92	89	0.595	92	98

Abbreviations: WHO, World Health Organization; ECOG, Eastern Cooperative Oncology Group; 2D-RT, Two-dimensional radiotherapy; IMRT, Intensity-modulated radiotherapy; DM, distant metastasis.

a The 7th AJCC/UICC staging system.

b *P* value indicates the difference between pregnant group and non-pregnant group.

Among the cohort of non-pregnant patients, 51 patients were selected according to the hierarchy selection criteria to establish the randomly assigned matches. Of the 51 pairs, 43 pairs were exactly matched. Of the remaining 8 pairs that were not exactly matched, two patients in the non-pregnant group had larger T stages than the matched pregnancy-associated patients (T2N2 vs. T1N2 and T2N3 vs. T1N3), and one patient in the non-pregnant group had a larger N stage than the matched pregnancy-associated patient (T2N1 vs. T2N0). One patient in the pregnancy-associated group received CRT along with IMRT, whereas the matched non-pregnant patient received only RT alone. There were 3 and 1 patients in the non-pregnant group who were not matched with the pregnancy-associated group regarding the year of diagnosis and the examination regimen, respectively.

### Survival analysis

**Figure 2:**
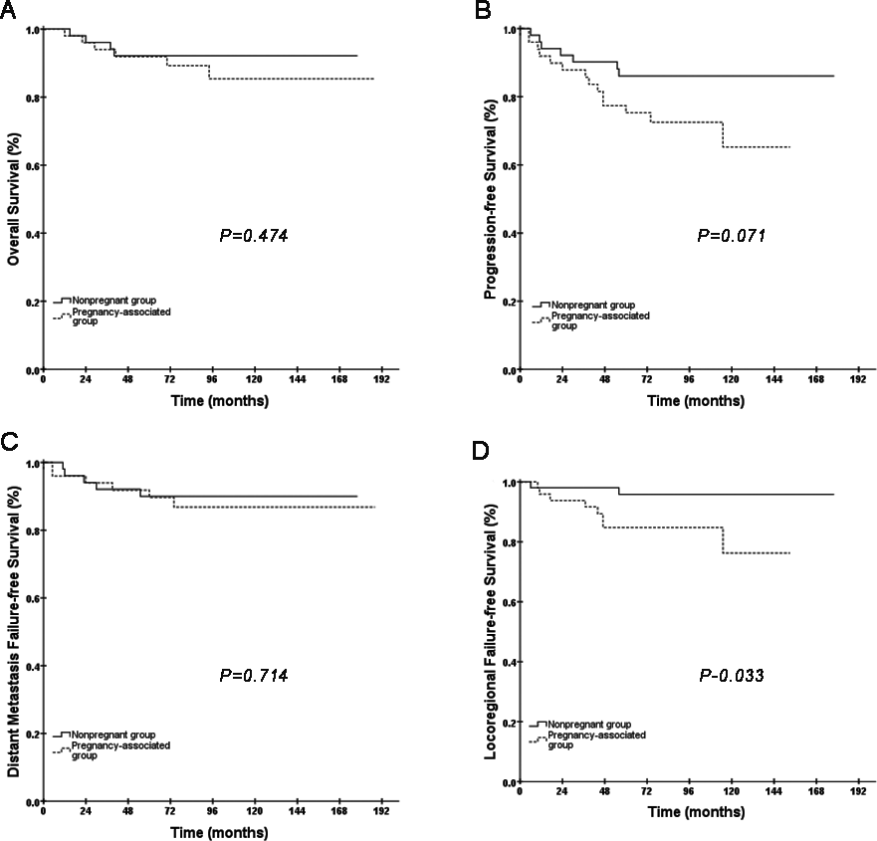
Kaplan-Meier curves of OS, PFS, DMFS and LRFS for the total group of female patients (non-pregnant group and pregnancy-associated group)

At the median follow-up time of 92 months (range 13–188), 6 (11.8%) patients in the pregnancy-associated group had died, 8 (16%) had locoregional relapse and 6 (11.8%) had distant failure. In the non-pregnant group, 4 (7.8%) patients had died, 2 (4%) had locoregional relapse and 5 (9.8%) had distant failure. After treatment, 10 patients in the pregnancy-associated group had PR, and 5 patients in the non-pregnant group had PR; the others were recorded as CR. A comparison of the pregnancy-associated group with the non-pregnant group showed a pattern similar to the pregnancy patients. A direct comparison of OS between the pregnancy-associated group and the non-pregnant group suggested that survival in the pregnancy-associated group was not compromised compared to that in the non-pregnant group (85.4% vs. 92.2%, *P* = 0.478, Figure 2A) with a hazard ratio of 1.582 (95% CI: 0.446–5.606). We did not record a difference in DMFS either (Figure 2C). The difference in PFS between the two groups was marginal (*P* = 0.071, Figure 2B). We found evidence of decreased LRFS for women in the pregnancy-associated group compared to patients in the non-pregnant group (84.8% vs. 95.9%, *P* = 0.033, Figure 2D).

### Effect of the pregnancy interval on the pregnancy-associated group and the non-pregnant group

**Figure 3:**
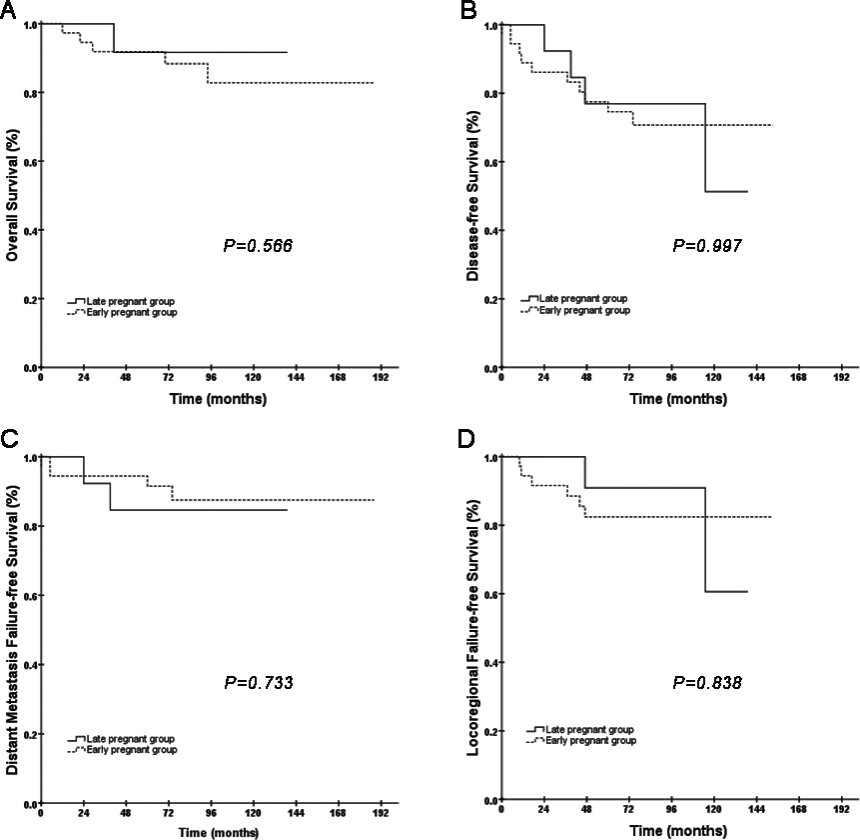
Kaplan-Meier curves of OS, PFS, DMFS and LRFS comparing the difference between the early pregnancy group and the late pregnancy group

The pregnant women were stratified into two subgroups: the “early pregnancy group”, which was defined as the patients who were diagnosed with NPC during pregnancy or lactation (6 months after delivery), and the “late pregnancy group”, which was defined as the patients who were diagnosed with NPC at least 6 months after pregnancy but within one year. Then, we evaluated whether the early pregnancy group and the late pregnancy group had a significantly different survival. Of the 51 pregnancy-associated women, 37 were assigned into the early pregnancy group and 14 were placed into the late pregnancy group. We did not find any differences in OS, PFS, DMFS or LRFS between these two subgroups (Figure 3A–3D).

**Figure 4:**
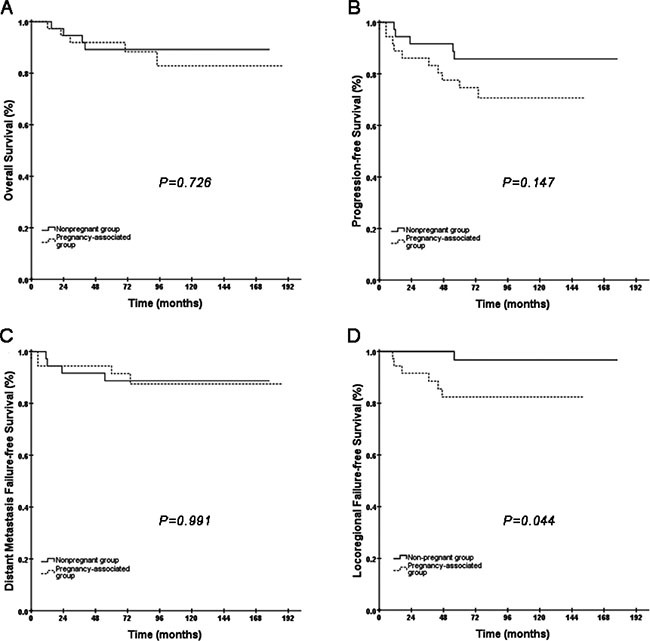
Kaplan-Meier curves of OS, PFS, DMFS and LRFS comparing the difference between the early pregnancy group and the matched non-pregnant group

**Figure 5:**
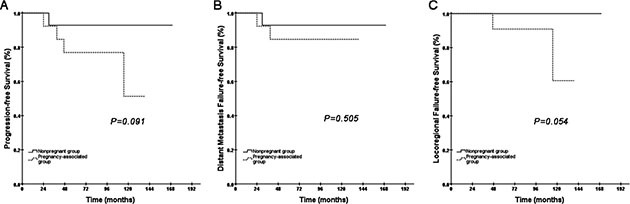
Kaplan-Meier curves of PFS, DMFS and LRFS for the late pregnancy group and the matched non-pregnant group Because only one patient died in the late pregnancy group and no one died in the matched non-pregnant group, the Kaplan-Meier OS curve could not be established.

Analyses of patients in the early pregnancy group and the matched patients revealed that no statistically significant different results were noted in OS, PFS or DMFS (Figure 4A–4C). However, LRFS for the patients in the early pregnancy group was inferior compared to the matched patients (82.4% vs. 96.8%, *P* = 0.044, Figure 4D). Conversely, in the late pregnancy group and the matched group, the results for OS, PFS, LRFS and DMFS suggested no significant difference between the two groups (Figure 5A–5D). Among the patients, we recorded a borderline *P* value for LRFS between the late pregnancy group and the corresponding matched group (*P* = 0.054, Figure 5C).

## DISCUSSION

In this study, our data suggested that the overall survival of pregnant NPC patients was not worse than non-pregnant NPC patients, and pregnancy did not appear to be a risk factor of death for NPC patients. This phenomenon was quite different from a very early study [1] conducted in 1983 by Yan et al. In Yan et al's study, only 27 patients were admitted into the study, and they were divided into two groups (a concurrent group with 9 women and a subsequent group with 18 women). The former group reported a disastrous effect on the prognosis of NPC patients with a 5-year survival of only 11% (1/9), whereas this adverse outcome was not observed in the latter group. At that time, the investigators were unsure of the real mechanisms by which pregnancy influences the prognosis of NPC, but they questioned whether the physiological anemia and depression of immunity during pregnancy might cause failure of local control and the development of distant metastases. However, one caveat must be noted: in the study by Yan et al. [1] a concurrent pregnant group (discovered to be pregnant during their treatment) and a subsequent group (became pregnant during their follow-up after radiotherapy) were used, whereas in our study we defined pregnancy-associated patients as women who were diagnosed with NPC during pregnancy or within one year after the completion of delivery. For other tumors, such as being pregnant at the diagnosis of breast cancer, a worse survival outcome may be predicted. Breast cancer, which was frequently diagnosed during pregnancy, has two findings in recent years: pregnancy was a protective factor for pregnant patients with breast cancer, and if patients were pregnant at the diagnosis of breast cancer, a worse survival outcome could be predicted. Amant et al. [5] registered breast cancer patients who were diagnosed during and within 1 year after pregnancy; this was called pregnancy-associated breast cancer. In that study, they estimated the prognostic effect of pregnancy when breast cancer was diagnosed, the results showed a similar OS for pregnant patients with breast cancer compared to non-pregnant patients with HR = 1.19 (95% CI: 0.73 to 1.93; *P* = 0.51). However, in their study, they did not stratify patients according to different time intervals. Another study on breast cancer by Hatem et al. [6] demonstrated that no difference in PFS was observed between pregnant and non-pregnant patients in the ER-positive (HR = 0.91; 95% CI: 0.67 to 1.24, *P* = 0.55) or ER-negative (HR = 0.75; 95% CI: 0.51 to 1.08, *P* = 0.12) cohorts. However, the pregnant group had a better OS (HR = 0.72; 95% CI: 0.54 to 0.97, *P* = 0.03) with no interaction according to ER status (*P* = 0.11). Pregnancy outcome and BC–pregnancy interval did not appear to affect the risk of relapse. Other cancers, such as cutaneous malignant melanoma in Marko et al's study, demonstrated similar results in that the survival of pregnant women with melanoma was not worse than that of non-pregnant women with melanoma [7]. We suspect that there may be some beneficial effect that is unexplained for pregnant patients with NPC or other cancers. The findings in our current study will help us inform such populations of how their disease may develop if they experience these circumstances in the future.

To the best of our knowledge, we addressed for the first time the prognostic effect of the time interval in NPC patients between pregnancy and the matched non-pregnant patients. No previous matched cohort analysis had shown convincing evidence regarding the role of the time interval. In a recently published study by Chen et al. [4], case-control analysis was used and similar survival outcomes were obtained except the loco-regional relapse-free survival (LRRFS). Their results demonstrated that the 5-year survival rates of OS, DMFS, LRRFS and disease-free survival (DFS) between the pregnancy-associated nasopharyngeal carcinoma (PANPC) were not significantly different. But we should note that our matching work was stricter than Chen el al's. In their study, they just adjusted for age, stage and chemotherapy mode while we completed the matching work in a descending hierarchy including 9 factors of nodal status, tumor size, PS, histology, radiation technique, treatment strategy, age at diagnosis, year of diagnosis and staging method. In addition, we had a relatively larger population of pregnant patients (51 patient vs. 36 patients), longer follow-up duration (92 months vs. 70 months) and focused on one particular group of young female (≤ 35 years) which may be of importance in guiding the management of such patients. Furthermore, our current study revealed that stages in the non-pregnant group and pregnant group before matching were comparable. Moreover, our subgroup analyses determined that in the early pregnancy group, a slightly favorable effect of LRFS was observed in the non-pregnant patients (*P* = 0.044). Likewise, we found a marginal effect of LRFS (*P* = 0.054) in the late pregnancy group. Hence, pregnancy may be a risk factor of locoregional control for pregnant patients. However, we did not have sufficient data to fully explain this phenomenon. Maybe just like what previous physiological studies have stated, depression in the immune system during pregnancy may cause a failure in local control. Of note, our study did not have adequate power to provide a definitive answer for these hypotheses, and we lack the biological material to test which biomarkers were higher or lower during pregnancy. For example, because of the time span of this matched cohort analysis study, we were able to obtain EBV DNA values for only 7 pairs in the pregnancy-associated group and no pairs in non-pregnant group; thus, we could not calculate the effect of EBV DNA on patients.

Our study has several strengths and limitations. Our main strength is that we used a matched method to minimize selection bias. However, all the cases were reported retrospectively, and no such matched cohort study exists from which NPC can be selected and in a particular order. Of course, we could not eliminate the effect of interruption on pregnant patients in advance. For example, during clinical treatment, doctors may pay more attention to pregnant women and perform more aggressive regimens on those women, such as active nutrition support or even blood transfusion when the hemoglobin level or red blood cells were not lower than the lower limit value. In addition, our matched cohort study had a small sample size, and we did not have enough serum or nasopharyngeal tissue samples for testing the tumor markers of pregnant patients compared to non-pregnant patients. Finally, most of our patients were treated with 2D-RT; thus, we are unsure whether pregnant patients with NPC could have a better outcome with IMRT, which is currently used frequently.

## CONCLUSIONS

In conclusion, this study indicated that pregnancy was not a risk factor for the survival of patients with NPC.

## PATIENTS AND METHODS

### Study design

We designed a retrospective matched cohort study of all female patients who were diagnosed with NPC at their reproductive age of ≤ 35 years from June 1999 to December 2010. Patients who became pregnant during their NPC diagnosis or within one year of the diagnosis were admitted as the pregnancy-associated group. Female patients who were not pregnant at diagnosis were admitted as the non-pregnant group. First, we selected potentially eligible subjects according to the following criteria: (1) female, (2) diagnosed with first primary NPC at a young age (defined as between 15 to 35 years old), (3) histology was shown to be World Health Organization (WHO) type I to type III, and (4) received treatment of chemoradiotherapy (CRT) or radiotherapy (RT) alone. Patients who had received previous treatment were excluded. Next, we compared baseline patient characteristics between the pregnancy-associated group and the enrolled non-pregnant group. Then, we excluded the patients who were diagnosed with distant metastasis at the initial staging. Finally, we matched female patients in the pregnancy-associated group with patients from the non-pregnant group.

The matching was performed within our cancer center, and the aim was to obtain one non-pregnant patient for each pregnancy-associated patient. To investigate the independent effect of pregnancy on survival outcomes, we attempted to complete the matching work according to the following factors in a descending hierarchy: nodal status, tumor size, performance status (PS) (0–1 or 2), histology, radiation technique [two-dimensional radiotherapy (2D-RT) or intensity-modulated radiotherapy (IMRT)], treatment strategy (CRT or RT alone), age at diagnosis, year of diagnosis (1999–2005 or 2006–2010) and staging method [computed tomography (CT) or magnetic resonance image (MRI)]. If an exact matched patient was unavailable, the matching criteria were relaxed until one was found. We attempted to select a non-pregnant patient with a poorer prognosis, for example: (1) older age, (2) more advanced nodal (N) stage, and (3) larger tumor (T) size. In our study, the most frequent reason for the inability to find an exact match was age; thus, an age difference between two patients of up to 5 years was used. This study was approved by the Ethics Committee of Sun Yat-sen University Cancer Center.

### Clinical assessment

All the patients underwent a pretreatment evaluation including a detailed history of disease, a physical examination of the head and neck, hematology and chemistry profiles, nasopharyngeal endoscopy, MRI and/or CT, chest X-ray, abdominal ultrasound, a whole-body bone scan, and a dental evaluation. Patients who had lower positive nodes received chest CT. PET/CT was available if economy permitted. Because most patients were staged using the 5th or 6th edition of the American Joint Committee on Cancer/Union for International Cancer Control (AJCC/UICC) system, we then reclassified the stages using the 7th edition of the AJCC/UICC.

### Treatment

Because treatment modalities evolved during the course of this study, the recommended treatment differed based on the date of diagnosis. Details about the RT technique that was conducted at our cancer center have been reported previously [8–10]. Conventional external-beam radiation therapy was delivered in a total dose ranging from 70–76 Gy to the primary lesion, 50–60 Gy to the upper neck and 50–55 Gy to the lower neck (including the supraclavicular region). The involved lymph nodes were boosted to a total dose of 65–70 Gy. The prescribed dose of IMRT was 68–70 Gy at 2.19–2.33 Gy/fraction over30–32 fractions to the PTVnx, 60–66 Gy/30–32 fractions to the positive neck lymph nodes for therapeutic intent, 60 Gy/30–32 fractions to the CTV1, and 54 Gy/30–32 fractions to the CTV2. The irradiation was delivered daily from Monday to Friday during each treatment week for 6–7 weeks at our cancer center.

The patients receiving CRT underwent concurrent cisplatin-based chemotherapy with/without sequential chemotherapy (induction and/or adjuvant). The ultimate decisions regarding the RT technique and the use of chemotherapy were based on the clinical stage, the physician's discretion and the patient's choice.

### Patient evaluation and follow-up

When the treatment ended, the responses at the primary and lymph node sites were evaluated by CT or MRI of the head and neck and with nasopharyngoscopy. The evaluations were conducted according to the Response Evaluation Criteria in Solid Tumor (RECIST, version 1.1). If the tumor remained when radiotherapy ended, examinations were repeated to evaluate the patient again 3 months later. After, all the patients were assessed once every 3 months over the next 3 years and then 6 months thereafter. In the follow-up visits, endoscopy, a physical examination, basic chemical profiles, chest X-ray, abdominal ultrasound and head and neck MRI were performed every 6 months. A bone scan and CT of the chest or abdomen and even PET/CT were performed when clinically indicated for the patient.

### Data statistics

The primary outcome was overall survival (OS), which was defined as the time from the date of treatment to the death from any cause or the date of the last follow-up visit. The secondary outcomes included progression-free survival (PFS), which was defined as the time from the date of treatment to any observation of disease progression including death or the date of the last follow-up, distant metastases failure-free survival (DMFS), which was defined as the time from the date of treatment to any observation of metastatic lesions or the date of the last follow-up, and locoregional failure-free survival (LRFS), which was defined as the time from the date of treatment to any observation of local or regional failure or the date of the last follow-up.

The categorical variables were compared using Pearson's *χ*^2^ test. Survival plots for the pregnancy-associated group and the non-pregnant group were created using the Kaplan-Meier method, and the differences were evaluated using the log-rank test. The reported *P* values were two-sided, and *P* values less than 0.05 were considered statistically significant. The statistical analyses were performed using SPSS, version 17.0 (SPSS, Chicago, IL, USA).
